# Expression patterns of flowering genes in leaves of ‘Pineapple’ sweet orange [*Citrus sinensis* (L.) Osbeck] and pummelo (*Citrus grandis* Osb*eck*)

**DOI:** 10.1186/s12870-017-1094-3

**Published:** 2017-08-30

**Authors:** Melanie Pajon, Vicente J. Febres, Gloria A. Moore

**Affiliations:** 1 Horticultural Sciences Department, Institute of Food and Agricultural Sciences, University of Florida, 2550 Hull Road, Gainesville, FL 32611 USA; 2 Plant Molecular and Cellular Biology Program, University of Florida, Gainesville, FL 32611 USA

**Keywords:** Florigen, *FLOWERING LOCUS T*, Flowering periodicity, Gene expression, Juvenility, Time course

## Abstract

**Background:**

In citrus the transition from juvenility to mature phase is marked by the capability of a tree to flower and fruit consistently. The long period of juvenility in citrus severely impedes the use of genetic based strategies to improve fruit quality, disease resistance, and responses to abiotic environmental factors. One of the genes whose expression signals flower development in many plant species is *FLOWERING LOCUS T (FT)*.

**Results:**

In this study, gene expression levels of flowering genes *CiFT1, CiFT2 and CiFT3* were determined using reverse-transcription quantitative real-time PCR in citrus trees over a 1 year period in Florida. Distinct genotypes of citrus trees of different ages were used. In mature trees of pummelo (*Citrus grandis* Osbeck) and ‘Pineapple’ sweet orange (*Citrus sinensis* (L.) Osbeck) the expression of all three *CiFT* genes was coordinated and significantly higher in April, after flowering was over, regardless of whether they were in the greenhouse or in the field. Interestingly, immature ‘Pineapple’ seedlings showed significantly high levels of *CiFT3* expression in April and June, while *CiFT1* and *CiFT2* were highest in June, and hence their expression induction was not simultaneous as in mature plants.

**Conclusions:**

In mature citrus trees the induction of *CiFTs* expression in leaves occurs at the end of spring and after flowering has taken place suggesting it is not associated with dormancy interruption and further flower bud development but is probably involved with shoot apex differentiation and flower bud determination. *CiFTs* were also seasonally induced in immature seedlings, indicating that additional factors must be suppressing flowering induction and their expression has other functions.

**Electronic supplementary material:**

The online version of this article (10.1186/s12870-017-1094-3) contains supplementary material, which is available to authorized users.

## Background

Many *Citrus* species are characterized as having extended juvenility periods and therefore not producing flowers or fruit for many years, up to a decade or longer [1, 2]. The process that leads to flowering in citrus trees most likely involves environmental and physiological cues. In *Arabidopsis thaliana* flowering cues include phytohormones such as gibberellic acid (GA), vernalization, light, gene expression patterns and other physiological responses [3]. Floral development occurs in the shoot apical meristem; however, some of the environmental response pathways that are involved act in the leaves. *Arabidopsis* plants are known to remain in the vegetative state under short day conditions- 8 or 10 h of light. When shifted to long days-16 h of light- genes that are involved in the flowering process are expressed in the meristem within 24 h [4]. Several studies have also demonstrated the importance of a low-temperature condition in the induction of flowering in citrus [5, 6]. This temperature condition has been shown to have an effect on the seasonal periodicity of flowering, yet there are few studies of the effect that this and other environmental cues have on the expression of genes in the flowering pathways in citrus. In *Arabidopsis* many genes involved in the flowering pathways have been extensively studied and their roles are clearly understood [4]. The homologues of these flowering genes in perennial trees such as *Citrus* provide the foundation to further explore why the juvenility period among citrus species differs so greatly and to what extent seasonality affects genetic expression profiles.

Commercially important citrus types thrive in humid subtropical regions of the world such as Florida, Central China, Brazil and Mexico [2]. These regions have a balance of rainfall, sunlight, wind, humidity, and temperature that favors the growth and production of citrus. It is therefore important to understand how factors in these particular regions affect seasonal periodicity and consequently gene expression for flowering pathway genes. Florida in particular has high temperatures from April to October; although summer temperatures rarely exceed 40 °C. During the summer, low temperatures range from near 21 °C in northern Florida to near 27 °C in the Keys, at the southern end of the state. High temperatures during the summer average 33 to 35 °C statewide. Moderate to severe freezes do occur in Florida between November and March but the climatic conditions in this state are optimal for certain commercial citrus types [2]. Economically important citrus cultivars such as ‘Pineapple’ sweet orange (*Citrus sinensis* (L.) Osbeck) and pummelo (*Citrus grandis* Osbeck) are known to have long juvenility periods and seasonal flowering in Florida, underscoring that these are two separate but related processes. For these species of citrus, molecular mechanisms involved in the onset of flowering have yet to be characterized. Indeed, most such studies to date have been conducted in mandarins in Japan under climatic conditions that are quite different from those in Florida [6].

Perhaps the most widely studied flowering gene across a variety of genera is *FLOWERING LOCUS T* (*FT*). In *Arabidopsis* expression of the *FT* gene and the *SUPPRESSOR OF OVEREXPRESSION OF CONSTANS 1 (SOC1)* gene are believed to be required for *CONSTANS (CO)* to induce flowering. *FT* is typically maximally expressed at the onset of floral induction [7]. Constitutive expression of the *Arabidopsis FT* gene in apple [8], soybean [9], and poplar [10] results in early flowering phenotypes and the expression levels of FT in Satsuma mandarin (*Citrus unshiu* Marc.) have been correlated with flower numbers [11]. Additionally, *FT* orthologues have been identified in a variety of genera such as rice [12], poplar [13, 14], citrus [6, 15], and many others. In citrus, the *CiFT1* homologue from Satsuma mandarin [6] was constitutively expressed in trifoliate orange and conferred a notably early-flowering phenotype [15]. Viral vectors based on *Citrus leaf blotch virus* have also been used to constitutively express *A. thaliana* and *C. sinensis FT* genes and were capable of inducing flowering in juvenile plants of a variety of citrus types [16].

Nishikawa et al. [6] conducted a quantitative real-time PCR study in Japan that included *CiFT1* and two novel homologues of *FT* in citrus (*CiFT2 and CiFT3*), although *CiFT1* and *CiFT2* are proposed to be alleles of the same locus [17]. This study provided some insight into expression patterns for several genes involved in flowering activity, yet no conclusive patterns were established for citrus trees under field conditions. Overall, some of the pathways and mechanisms of flowering in *Arabidopsis* have been shown to be conserved in other species including woody perennials, yet more research must be done to investigate specific molecular mechanisms in *Citrus*.

To summarize, for flowering to occur in citrus two events are needed: 1) plants must reach maturity and 2) they have to be exposed to the right environmental cues. Hence the main objective of the present study was to compare the seasonal periodicity of the FTs gene expression in leaves of mature and juvenile citrus. Since FT is purportedly a major determinant of flowering induction in plants and flowering seasonality is observed under Florida’s conditions we set out to investigate how different its expression patterns were between mature (flowering) and juvenile (non-flowering) individuals. Furthermore, since citrus has at least two FTs, are there differences in expression levels that could indicate separate functions by age or season? Thus a 1 year study of in vivo tracking of *C*i*FT1, CiFT2* and *CiFT3* expression in various citrus trees differing in age and genotype was undertaken. Gene expression levels were compared on a month-to-month basis using the comparative C_T_ method of quantitative real time PCR. Leaf tissue was used for this purpose for several reasons. First, *FT* gene expression is known to occur in leaves while FT protein is translocated to the meristems [18]. Also, some of the trees analyzed were juvenile and did not produce flowers. Citrus, like many other perennial plants, has leaf and flower meristems in the same flushing stems and these different meristems cannot be easily distinguished, thus analyzing meristematic tissue, although a possibility, would have been problematic. Leaves as the examined tissue seemed appropriate since our purpose was not to compare different tissues but rather to measure gene expression as it related to the time of year and age. Thus, we looked at the production of transcripts from the selected genes throughout the year, not just at flowering.

## Methods

### Plant material

The citrus genotypes used in this study were ‘Pineapple’ sweet orange (PSO) (*Citrus sinensis* (L.) Osbeck) and pummelo (*Citrus grandis* Osbeck) (PUM). Two adult ‘Pineapple’ sweet orange trees of approximately 15 years of age were located in the USDA A. H. Whitmore Farm (23,402 USDA Rd., Groveland, FL 34736 (28°41′16.1″N 81°53′09.6″W; summer solstice daylight: 13:57 h, winter solstice daylight: 10:15 h; January average high: 22 °C, average low: 9 °C; July average high: 34 °C, average low: 23 °C). The temperatures registered during the experimental period are shown in Additional file 1: Figure S1. One of the sweet orange trees was a seedless mutant and the other had seed (“seedy”). An adult pummelo of approximately 15 years of age was located in a temperature-controlled greenhouse at the University of Florida, Gainesville, FL (29°38′20.8″N 82°21′35.6″W; summer solstice daylight: 14:03 h, winter solstice daylight: 10:14 h), the temperature set to range between 18 and 35 °C with no light supplementation. Three 2-year-old PSO trees were also in the same greenhouse. Therefore a total of 6 trees were used in this study. Leaves were collected from each tree once a month for 12 months on the same day from both locations. The year collection period began on 3 July 2012 and ended on 28 June 2013. For the PSO seedless and seedy and pummelo mature trees, three different leaf samples were collected from different parts of the trees and used as biological replicates. A representative sample for each replicate included multiple leaves ranging from new growth to older growth. For the 2-year-old PSO trees, three different plants were used as biological replicates and each sample also consisted of several leaves representative of the whole tree. Collected tissue was stored in aluminum foil in a cooler with dry ice during transport to a − 80 °C freezer in the laboratory.

### RNA extraction and cDNA synthesis

Total RNA was extracted using TriZol reagent (Invitrogen) according to the manufacturer’s instructions followed by DNase treatment and clean up with the RNeasy Plant Mini Kit (QIAGEN). The RNA concentration and purity were determined using a NanoDrop 2000c spectrophotometer (Thermo Scientific). A criterion was employed based on OD_260_/OD_230_ (≥ 1.7), OD_260_/OD_280_ (≥ 1.7) and RNA absorbance curves to deem any sample of acceptable quality. cDNA synthesis reactions were performed as follows: a total of 16 μL consisting of 1 μg of the purified RNA, 2 μL of 50 μM random decamers (Ambion), 2 μL of dNTPs (5mM each)  and RNase-free water were incubated at 80 °C for 3 min in a PTC-100 Programmable Thermal Controller (MJ Research Inc.) and then placed on ice for 3 min. Subsequently, a mixture of 1 μL (200 units) of M-MLV reverse transcriptase (Ambion), 2 μL of 10X First Strand Buffer and 1 μL (40 units) of RNase Inhibitor (Ambion) were added to the 16 μL sample for a final volume of 20 μL. The 20 μL reaction mixture underwent reverse transcription in the thermal cycler (PTC-100 Programmable Thermal Controller, MJ Research Inc.) using the following parameters: 42 °C for 1 h followed by 92 °C for 10 min. The cDNA product was stored at −20 °C. A final working 1:10 dilution was made from the 50 ng/μL cDNA stock.

### Gene expression analysis

Gene expression levels were measured with reverse-transcription quantitative real-time PCR (RT-qPCR) using the StepOnePlus Real-Time PCR system (Applied Biosystems). The parameters for reactions were set as follows: comparative C_T_ (ΔΔC_T_) with the fast amplification of 95 °C for 20s, 40 cycles of 95 °C for 1 s, and 60 °C for 20s. A Fast 96-well Reaction Plate (0.1 mL) (MicroAmp, Applied Biosystems) was used for the reactions and each well was used to perform a 20 μL expression assay of one gene per each sample. Gene amplification was performed with 10 ng of working cDNA solution. Each reaction mixture was composed of 2 μL of cDNA, 10 μL of TaqMan Fast Universal PCR Master Mix (2X) (Applied Biosystems), 1 μL of TaqMan probe and primer Assay Mix (20X) (Applied Biosystems) and 7 μL of RNase-free water. The 20X Assay Mix was a combination of specific TaqMan MGB probes, forward and reverse primers for each gene. For all of the assayed citrus flowering genes, the final primer concentration was 900 nM each and the final probe concentration was 250 nM. Probes were labeled with 6-carboxyfluorescein (FAM). For the endogenous control reference gene 5.8S rRNA was used, with a final primer concentration of 250 nM each and final probe concentration of 150 nM per reaction. The probe for 5.8S rRNA was labeled with 4,7,2′-trichloro-7′-phenyl-6-carboxyfluorescein (VIC). All primer and probe sequences, and sources are listed in Additional file 2: Table S1. Primers and probes for *5.8S rRNA* were designed with Primer Express Software (Applied Biosystems). The primers and probes used *for CiFT1, CiFT2* and *CiFT3* were taken from Nishikawa et al. [6]. A negative control containing all RT-qPCR reaction elements and water instead of cDNA was used for every 96-well reaction plate.

### Statistical analysis

For the comparative C_T_ analysis, the PSO seedy sample from 3 July 2012 (PSOsd M01-1) was used as the reference sample for PSO seedy and seedless 15-year-old trees and for the PSO 2-year-old trees. The pummelo sample from 3 July 2012 (PUM M01-1) was used as the reference sample for the pummelo genotype. Quantitative real-time PCR amplification data from twelve different time points and three biological replicates for each gene was normalized with the 5.8S rRNA C_T_ values and the threshold was automatically set but adjusted manually when needed. The relative quantitation (RQ) values were calculated using the StepOne software version 2.3 (Applied Biosystems) and exported to Microsoft Office Excel for further analysis. Outliers were identified using Dixon’s Q test at 95% confidence [19] and the quantile range outliers tool from JMP Genomics 8.2 (SAS Institute Inc. NC). The RQ data was used to calculate means and standard errors (*n* = 3 for PUM and 2-year-old PSO, *n* = 6 for 15-year-old PSO). Statistical analysis was performed using JMP Genomics model fitting of standard least square means (LS Means) and Student’s t test (*P* < 0.05) (Additional file 3. Table S2).

## Results and discussion

To investigate the expression patterns of genes involved in the flowering process of young and mature citrus trees in Florida, mRNA was extracted from representative samples of different species and ages and used in a quantitative real-time polymerase chain reaction (RT-qPCR) study. Expression levels were determined according to genotype, age and time points. The time points were chosen so that they would be representative of the 12 months in a year. The method used to acquire three biological replicates was based on the availability of trees of the same genotype and age. Since no young trees existed at the Groveland, Florida location we used young, greenhouse trees located at our Gainesville, Florida campus.

All three *CiFT* and internal control genes chosen for the study were detected in the leaves collected for each sample at every time point. Leaves that ranged from new growth to old growth were selected from different parts of the trees in order to get a more representative profile for the entire tree.

### *CiFTs* expression in mature citrus

**Fig. 1:**
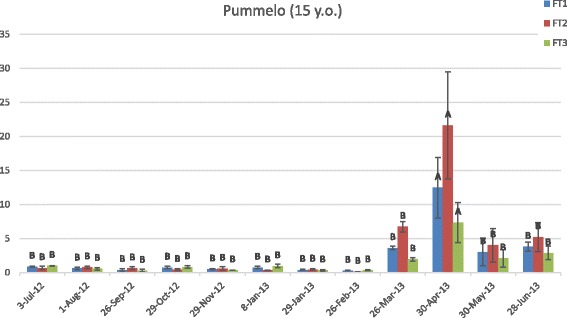
Expression of *C*i*FT1, CiFT2, and CiFT3* in leaves of pummelo. Gene expression was quantified throughout a 12-month period by real-time PCR and evaluated using the comparative CT analysis. The vertical axis indicates the relative quantitation (RQ) of gene expression levels after each sample is compared to a reference sample from 3-Jul-12. The horizontal axis displays the collection dates. Data are means ±SE (*n* = 3). Levels A and B of the Student’s t analysis are indicated. Columns with different letters are significantly different

**Fig. 2:**
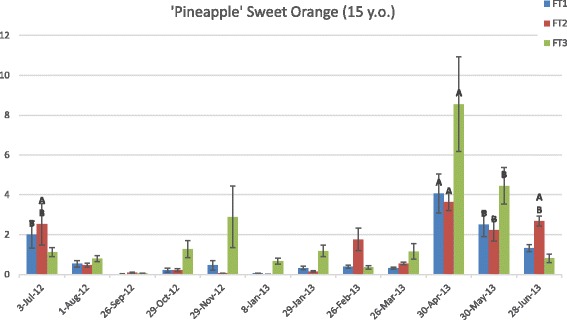
Expression of *CiFT1*, *CiFT2*, and *CiFT3* in leaves of adult ‘Pineapple’ sweet orange. Gene expression in 15-year-old trees was quantified throughout a 12-month period by real-time PCR and evaluated using the comparative CT analysis. The vertical axis indicates the relative quantitation (RQ) of gene expression levels after each sample is compared to a reference sample from 3-Jul-12. The horizontal axis displays the collection dates. Data are means ±SE (*n* = 6). Levels A and B of the Student’s t analysis are indicated. Columns with different letters are significantly different

In the mature pummelo tree, the mRNA levels for *CiFT1, CiFT2,* and *CiFT3* were highest on 30 April 2013 compared to all other time points (Fig. 1). In general, *CiFT* levels were highest between March and June, with *CiFT2* showing the highest transcript levels, followed by *CiFT1* and then *CiFT3*. In April 2013, the 15-year-old pummelo tree was bearing immature fruit after flowering events in late February and early March. Similarly, the mature PSO (Fig. 2) presented the highest levels of *CiFT* transcripts on 30 April 2013 with overall levels highest between April and June 2013 and July 2012. However, unlike Pummelo, *CiFT3* exhibited the highest transcription level (during April and May, Fig. 2) compared to *CiFT1*/*FT2* and all three *CiFT* genes reached lower relative expression levels compared to pummelo. By this time point in the field in Florida, bloom was largely over [20] and trees were bearing mature and immature fruit (the fruits were not harvested from the experimental trees). Hence, *C*i*FT1, CiFT2,* and *CiFT3* gene expression in leaves of mature plants was mostly synchronized and highest soon after flowering. This timing was surprising because in the field in Florida, flower bud induction, when signals are presumably sent to apical buds, occurs from roughly mid-October to the end of January and a release of cool temperatures is thought to be necessary for flower bud development, which in the study area occurred during the November 2012-March 2013 period, with the lowest minimum temperatures registered in March 2013 (Additional file 1: Figure S1). Furthermore, the gene expression profile for the mature pummelo grown in the greenhouse was similar to the ones seen in the field-grown PSO trees. It is worth mentioning that although the temperature in the greenhouse was controlled, it fluctuated between 18 and 35 °C throughout the year, remaining on the cooler side during the winter months (also November-March). It is possible then that, in addition to temperature, other environmental factors such as light conditions also function as cues for the regulation of *FT* expression. Another possibility is that leaf expression of *FTs* in Citrus is dependent on internal signals. The results also indicate that leaf expression of *CiFTs* is not associated with dormancy interruption and further flower bud development (which occurred long before the observed expression peak in April) but may be associated with shoot apex differentiation and flower bud determination.

### *CiFTs* expression in immature citrus

**Fig. 3:**
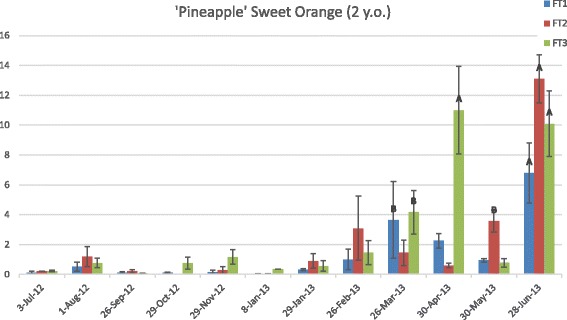
Expression of *C*i*FT1, CiFT2, and CiFT3* in leaves of young ‘Pineapple’ sweet orange trees. Gene expression in 2-year-old trees was quantified throughout a 12-month period by real-time PCR and evaluated using the comparative C_T_ analysis. The vertical axis indicates the relative quantitation (RQ) of gene expression levels after each sample is compared to a reference sample from 3-Jul-12. The horizontal axis displays the collection dates. Data are means ±SE (*n* = 3). Levels A and B of the Student’s t analysis are indicated. Columns with different letters are significantly different

Seasonality in the expression of the *CiFT* genes was also observed in seedlings. In immature PSO, *CiFT1* transcripts were highest on 28 June 2013 (Fig. 3). *CiFT2* expression was also highest on 28 June 2013, displaying the highest transcript level of all three *CiFTs*. On the other hand, *CiFT3* expression was highest on 30 April and 28 June 2013. Overall the PSO *CiFTs* displayed higher expression levels in immature plants than in mature trees, with fluctuations from March through June. These plants were not flowering so the function of these proteins is probably related to vegetative growth or some other function. In addition to having a role in flowering induction and breaking flowering dormancy FT seems to affect vegetative growth, leaf, flower and inflorescence architecture and thorn development [15, 21–24]. For instance overexpression of *FT* in *Poncirus trifoliata*, a citrus relative, alters tree architecture, dormancy requirements and leaf shape [15].

## Conclusions

In mature citrus trees there were no differences between the leaf expression patterns of the three *CiFT* genes studied. Their expression was synchronized, peaked around April and subsided in the following months. This pattern was observed in the trees regardless of whether they were under temperature controlled conditions in the greenhouse or in the field. This was also a major difference with juvenile plants in which *CiFT3* but not *CiFT1/FT2* was induced during 30 April 2013.

FT protein, produced in the leaves is transported to the apical meristems where, through its interaction with other proteins, it triggers the formation of floral meristems [18]. Furthermore, it has been observed in various systems, including citrus, that ectopic overexpression of *FT* overcomes juvenility and induces early flowering [10, 15, 16, 23, 25, 26]. Interestingly, we observed that in leaves of immature PSO plants FT expression was highly induced, particularly during the month of June, indicating that in juvenile wild type plants high expression levels of *FT* in leaves was not associated with flowering. Assuming *CiFT* mRNA levels correlate with FT protein levels and that it gets transported to the meristem then FT is not sufficient to induce flowering and it must be preceded or accompanied by superseding factors that enable the transition from juvenile to mature. This is perhaps an evolutionary strategy to guarantee the individual has reached the appropriate size or accumulated enough resources to provide the best chances for fructification and hence reproductive survival. It seems from these results that, at the expression levels observed in wild type individuals, *CiFT* expression is necessary but not sufficient to induce flowering. The transition from juvenile to mature must first happen for flowering to occur. This is not the case in transgenic plants, perhaps because exceptionally high levels of *FT* are reached. In fact a correlation between levels of expression and timing of flowering has been observed [15].

The induction of *FT* genes expression in citrus leaves coincided with the transition from winter to spring (Additional file 1, Figure S1) and it could have been triggered by temperature (either accumulation of cold hours or the change to warmer temperatures) or increasing day length or both.

## Additional files

Additional file 1: Figure S1. Temperatures registered during the experimental period in Groveland, Florida. (a) Monthly average minimum and maximum temperatures. (b) Monthly low and high temperature range. Source: Weather Warehouse (http://www.usclimatedata.com/climate/clermont/florida/united-states/usfl0086 and https://www.wunderground.com/weather/us/fl/clermont). (PDF 189 kb)

Additional file 2: Table S1. TaqMan MGB primers and probes. (PDF 136 kb)

Additional file 3: Table S2. Full Student’s t test results. (PDF 219 kb)

## Abbreviations

C_T_: Cycle threshold
mRNA: Messenger ribonucleic acid
PCR: Polymerase chain reaction
PSO: ‘Pineapple’ sweet orange
PSOsd: ‘Pineapple’ sweet orange, seedy
PUM: Pummelo
RQ: Relative quantification
rRNA: Ribosomal ribonucleic acid
RT-qPCR: Reverse-transcription quantitative real-time PCR
SE: Standard error
