# Supplementary figures and images for: Expression patterns of flowering genes in leaves of ‘Pineapple’ sweet orange [Citrus sinensis (L.) Osbeck] and pummelo (Citrus grandis Osbeck)
Source: BMC Plant Biol. 2017 Aug 30;17:146. doi: 10.1186/s12870-017-1094-3 (PMC5577756; doi:10.1186/s12870-017-1094-3)

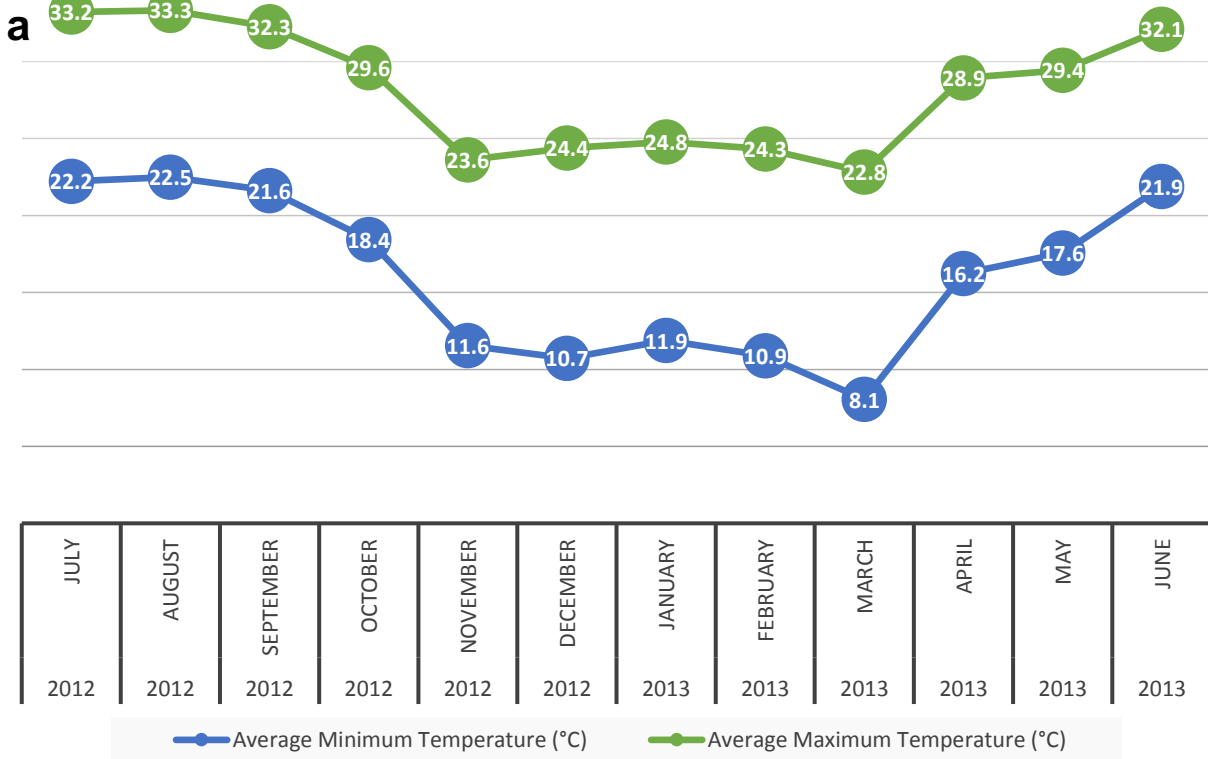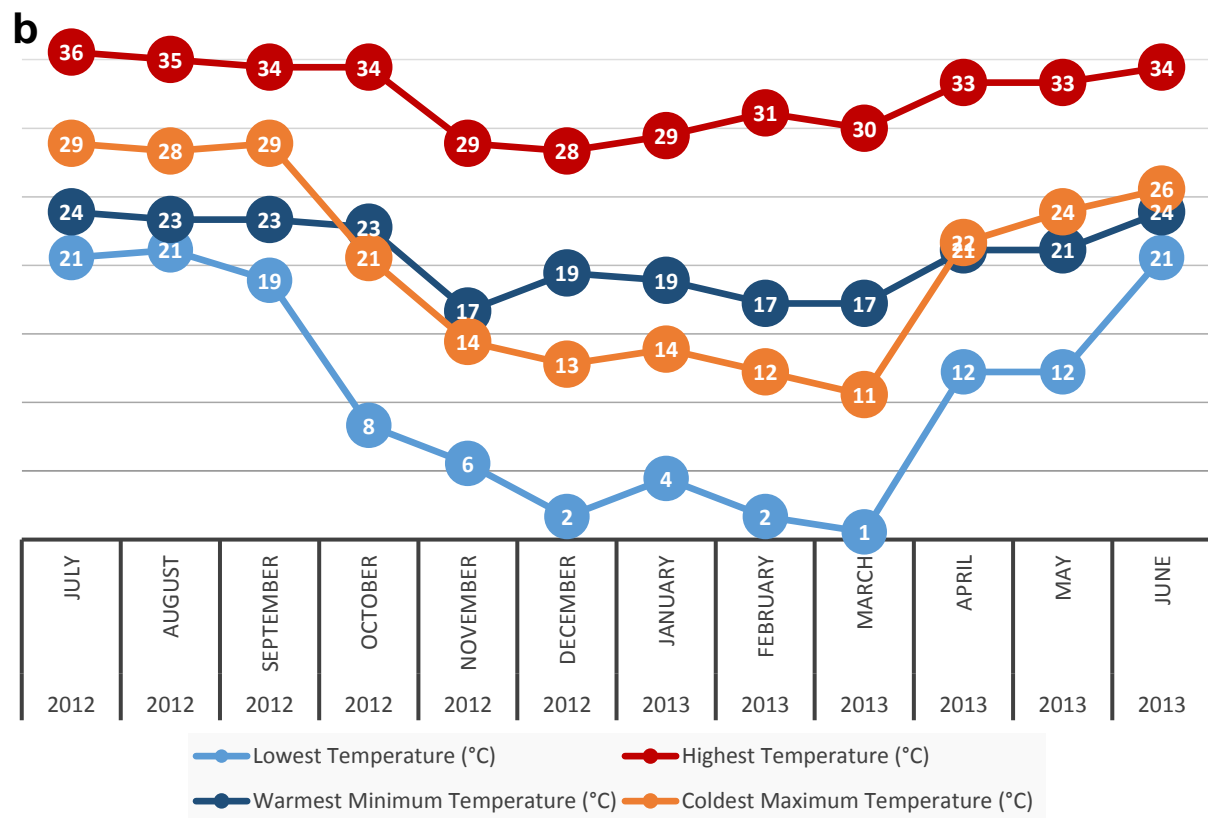

Supplement: Supplementary file 1 — Temperatures registered during the experimental period in Groveland, Florida. (a) Monthly average minimum and maximum temperatures. (b) Monthly low and high temperature range. Source: Weather Warehouse (http://www.usclimatedata.com/climate/clermont/florida/united-states/usfl0086 and https://www.wunderground.com/weather/us/fl/clermont). (PDF 189 kb) [file 12870_2017_1094_MOESM1_ESM.pdf]
