# Supplementary material for: Expression patterns of flowering genes in leaves of ‘Pineapple’ sweet orange [Citrus sinensis (L.) Osbeck] and pummelo (Citrus grandis Osbeck)
Source: BMC Plant Biol. 2017 Aug 30;17:146. doi: 10.1186/s12870-017-1094-3 (PMC5577756; doi:10.1186/s12870-017-1094-3)
Supplement: Supplementary file 2 — TaqMan MGB primers and probes. (PDF 136 kb) [file 12870_2017_1094_MOESM2_ESM.pdf]

Table S1. TaqMan MGB primers and probes.

**Target**	**Sequence (5' to 3')**
5.8S rRNA (*C. sinensis* JN681150.1; *C. grandis* JN681156.1)	Forward CGACTCTCGGCAACGGATA<br>Reverse CGCATTTCGCTACGTTCTTCA<br>Probe CTCGGCTCTCGCATC
CiFT1 (Nishikawa et al. 2007)	Forward CTACCAACAAAATTTCATCACTTGAATAG<br>Reverse GATCTCTCTCCCTGCTAGACATATCA<br>Probe TCTTACTACTTTTGTAGGCTGTGT
CiFT2 (Nishikawa et al. 2007)	Forward CTACCAACAAAATTTCATCACTTGAATAG<br>Reverse GGGTCTCTCTCCCTGCTAGACA<br>Probe TCTTACTACTTTTGTAGGTTGTTTG
CiFT3 (Nishikawa et al. 2007)	Forward CAACAAAATTTCATCACTTGAATAGTC<br>Reverse AAACACTCAACAACACTTAGCACAAA<br>Probe AGGCTGTGTGTGCGTGTA
