# Supplementary material for: Expression patterns of flowering genes in leaves of ‘Pineapple’ sweet orange [Citrus sinensis (L.) Osbeck] and pummelo (Citrus grandis Osbeck)
Source: BMC Plant Biol. 2017 Aug 30;17:146. doi: 10.1186/s12870-017-1094-3 (PMC5577756; doi:10.1186/s12870-017-1094-3)
Supplement: Supplementary file 3 — Full Student’s t test results. (PDF 219 kb) [file 12870_2017_1094_MOESM3_ESM.pdf]

Table S2. Full Student's t test results

Pummelo (15 y.o.)

Date	Letters	Least Sq Mean FT1	Std Error
3-Jul-12	B	0.88	1.43
1-Aug-12	B	0.61	1.43
26-Sep-12	B	0.39	1.43
29-Oct-12	B	0.75	1.43
29-Nov-12	B	0.47	1.43
8-Jan-13	B	0.74	1.43
29-Jan-13	B	0.39	1.43
26-Feb-13	B	0.28	1.43
26-Mar-13	B	3.62	1.43
30-Apr-13	A	12.45	1.43
30-May-13	B	3.01	1.43
28-Jun-13	B	3.80	1.43

Date	Letters	Least Sq Mean FT2	Std Error
3-Jul-12	B	0.68	2.47
1-Aug-12	B	0.77	2.47
26-Sep-12	B	0.62	2.47
29-Oct-12	B	0.39	2.47
29-Nov-12	B	0.60	2.47
8-Jan-13	B	0.32	2.47
29-Jan-13	B	0.45	2.47
26-Feb-13	B	0.11	2.47
26-Mar-13	B	6.72	2.47
30-Apr-13	A	21.61	2.47
30-May-13	B	3.99	2.47
28-Jun-13	B	5.21	2.47

Date	Letters	Least Sq Mean FT3	Std Error
3-Jul-12	B	0.98	0.99
1-Aug-12	B	0.56	0.99
26-Sep-12	B	0.28	0.99
29-Oct-12	B	0.82	0.99
29-Nov-12	B	0.34	0.99
8-Jan-13	B	0.95	0.99
29-Jan-13	B	0.33	0.99
26-Feb-13	B	0.35	0.99
26-Mar-13	B	1.94	0.99
30-Apr-13	A	7.35	0.99
30-May-13	B	2.10	0.99
28-Jun-13	B	2.87	0.99

'Pineapple' Sweet Orange (15 y.o.)

Date	Letters	Least Sq Mean FT1	Std Error
3-Jul-12	B	2.00	0.42
1-Aug-12	CD	0.53	0.42
26-Sep-12	D	0.02	0.42
29-Oct-12	CD	0.22	0.42
29-Nov-12	CD	0.46	0.42
8-Jan-13	CD	0.07	0.46
29-Jan-13	CD	0.32	0.42
26-Feb-13	CD	0.39	0.46
26-Mar-13	CD	0.31	0.46
30-Apr-13	A	4.06	0.42
30-May-13	B	2.51	0.42
28-Jun-13	BC	1.32	0.46

Date	Letters	Least Sq Mean FT2	Std Error
3-Jul-12	AB	2.52	0.42
1-Aug-12	D	0.46	0.42
26-Sep-12	D	0.08	0.42
29-Oct-12	D	0.22	0.42
29-Nov-12	D	0.05	0.42
8-Jan-13	D	0.01	0.46
29-Jan-13	D	0.14	0.42
26-Feb-13	BC	1.76	0.42
26-Mar-13	CD	0.55	0.46
30-Apr-13	A	3.64	0.46
30-May-13	B	2.22	0.42
28-Jun-13	AB	2.68	0.46

Date	Letters	Least Sq Mean FT3	Std Error
3-Jul-12	CD	1.12	0.90
1-Aug-12	CD	0.79	0.99
26-Sep-12	D	0.06	0.99
29-Oct-12	CD	1.27	0.90
29-Nov-12	BC	2.89	0.90
8-Jan-13	CD	0.67	0.90
29-Jan-13	CD	1.18	0.90
26-Feb-13	CD	0.36	0.90
26-Mar-13	CD	1.15	0.90
30-Apr-13	A	8.55	0.90
30-May-13	B	4.45	0.90
28-Jun-13	CD	0.81	0.90

'Pineapple' Sweet Orange (2 y.o.)

Date	Letters	Least Sq Mean FT1	Std Error
3-Jul-12	C	0.13	0.88
1-Aug-12	C	0.51	0.88
26-Sep-12	C	0.11	0.88
29-Oct-12	C	0.11	0.88
29-Nov-12	C	0.16	0.88
8-Jan-13	C	0.04	0.88
29-Jan-13	C	0.32	0.88
26-Feb-13	C	1.00	0.88
26-Mar-13	B	3.65	0.88
30-Apr-13	BC	2.25	0.88
30-May-13	C	0.94	0.88
28-Jun-13	A	6.80	1.07

Date	Letters	Least Sq Mean FT2	Std Error
3-Jul-12	D	0.21	1.01
1-Aug-12	BCD	1.20	0.83
26-Sep-12	D	0.21	0.83
29-Oct-12	D	0.01	0.83
29-Nov-12	D	0.28	0.83
8-Jan-13	D	0.05	0.83
29-Jan-13	CD	0.90	0.83
26-Feb-13	BC	3.09	0.83
26-Mar-13	BCD	1.45	0.83
30-Apr-13	D	0.60	0.83
30-May-13	B	3.57	0.83
28-Jun-13	A	13.09	1.01

Date	Letters	Least Sq Mean FT3	Std Error
3-Jul-12	C	0.23	1.18
1-Aug-12	BC	0.77	1.18
26-Sep-12	C	0.08	1.18
29-Oct-12	BC	0.75	1.18
29-Nov-12	BC	1.17	1.18
8-Jan-13	BC	0.35	1.45
29-Jan-13	BC	0.56	1.18
26-Feb-13	BC	1.47	1.18
26-Mar-13	B	4.17	1.45
30-Apr-13	A	11.01	1.18
30-May-13	BC	0.78	1.18
28-Jun-13	A	10.10	1.18

Levels not connected by the same letter are significantly different.
